# Evaluation of Efficacy of Water-Soluble Fraction of *Rhus semialata* Gall Extract and Penta-O-Galloyl-β-D-Glucose on Mitigation of Hair Loss: An In Vitro and Randomized Double-Blind Placebo-Controlled Clinical Study

**DOI:** 10.3390/antiox14040477

**Published:** 2025-04-16

**Authors:** Hee-Sung Lee, Jae Sang Han, Ji-Hyun Park, Min-Hyeok Lee, Yu-Jin Seo, Se Yeong Jeon, Hye Ryeong Hong, Miran Kim, Seon Gil Do, Bang Yeon Hwang, Chan-Su Park

**Affiliations:** 1 Department of Manufacturing Pharmacy, College of Pharmacy, Chungbuk National University, Cheongju 28160, Republic of Korea; sgboy5456@naver.com (H.-S.L.);; 2 Naturetech Co., Ltd., Cheonan 31257, Republic of Korea; 3 Unigen Inc., Cheonan 31257, Republic of Korea

**Keywords:** hair loss, hair dermal papilla cells, Penta-O-Galloyl-β-D-Glucose, water-soluble fraction of *Rhus semialata* gall extract

## Abstract

Hair loss, a prevalent condition affecting individuals across various demographics, is associated with hormonal imbalances, oxidative stress, inflammation, and environmental factors. This study evaluated the anti-hair loss potential of the water-soluble fraction of *Rhus semialata* gall extract (WRGE) and its primary component, Penta-O-Galloyl-β-D-Glucose (PGG), through both in vitro and clinical studies. WRGE was obtained using a standardized extraction process, and PGG was identified via HPLC-DAD and HRESIMS/MS techniques. Human dermal papilla cells (HDPCs) are specialized fibroblasts that can regulate the hair growth cycle and hair follicle growth. HDPCs are widely used in research focused on anti-hair loss. In this study, the anti-hair loss effects of WRGE and PGG on HDPCs were confirmed. WRGE and PGG enhance cell proliferation in HDPCs. These results are associated with the activation of the Wnt/β-catenin signaling pathway and the upregulation of hair growth factors such as vascular endothelial growth factor (VEGF), insulin-like growth factor-1 (IGF-1), and fibroblast growth factor (FGF). Furthermore, WRGE and PGG significantly inhibited dihydrotestosterone (DHT)-mediated DKK-1 secretion and H_2_O_2_-medicated cytotoxicity. Clinical trials further validated these results, demonstrating significant improvements in hair density and visual hair appearance scores in participants treated with WRGE compared to a placebo group. These results collectively suggest that WRGE and PGG may serve as promising natural agents for the prevention and treatment of hair loss by targeting multiple biological pathways, including the regulation of hair growth factors, oxidative stress, and hormonal imbalances.

## 1. Introduction

Hair loss is a common disorder that affects individuals of all ages and sexes [1]. It occurs due to various factors, including hormone-induced hair loss, reactive oxygen species (ROS)-induced damage, inflammation, drug treatments, aging, and environmental exposure [2,3]. Hair loss seriously affects people’s state of mind, leading to emotional distress, social anxiety, or depression [4,5]. Therefore, it is crucial to investigate new therapeutic agents for hair loss by elucidating the underlying mechanisms responsible for its occurrence.

The hair growth cycle is a continuous process transitioning from the anagen (growth phase) to the catagen (regression phase), and then to the telogen (resting phase), before returning to the anagen phase. When the anagen phase is shortened due to numerous factors, hair growth is inhibited, ultimately leading to hair loss. This cycle is regulated by hair follicles (HFs), which function as complex mini-organs involved in thermoregulation, sensory perception, and physical protection [6]. HFs are composed of multiple cell clusters and play a critical role between the epidermal and dermal compartments [7]. Human dermal papilla cells (HDPCs), specialized fibroblasts of hair follicles, regulate HF production and growth by secreting key hair growth factors, including vascular endothelial growth factor (VEGF), insulin-like growth factor (IGF-1), and fibroblast growth factor (FGF). The wingless (Wnt)/β-catenin signaling pathway regulates the expression of these growth factors in HDPCs. In patients with hair loss, a reduction in growth factor expression is observed, which is associated with the weakening of the Wnt/β-catenin signaling pathway in HDPCs [8,9]. Therefore, the regulation of HDPC activity is a critical factor in treating hair loss [10,11,12].

Dihydrotestosterone (DHT), a hormone elevated in androgenetic alopecia (AGA), inhibits the Wnt/β-catenin signaling pathway, contributing to hair loss [13]. DHT is converted from testosterone by 5α-reductase in HDPCs. In response to DHT stimulation, Dickkopf-related protein 1 (DKK-1) is secreted by HDPCs. DKK-1, a potent inhibitor of the Wnt/β-catenin pathway, disrupts the formation of the Wnt receptor complex and facilitates β-catenin degradation through a ubiquitin-dependent mechanism [14,15]. These actions negatively impact hair follicle morphogenesis and growth [16]. Elevated levels of DKK-1 in AGA patients further underscore its critical role in hair loss. Therefore, inhibiting DKK-1 and restoring the Wnt/β-catenin pathway represent promising therapeutic strategies for AGA.

Oxidative stress, caused by various factors, including aging, ultraviolet rays, and dietary habits, is another significant contributor to hair loss [17]. Oxidative stress induces the elevation of ROS in HDPCs, leading to DNA, protein, and lipid damage, while also inhibiting cellular repair mechanisms [16,18,19,20,21]. Moreover, HDPCs from balding scalps exhibit higher susceptibility to oxidative stress [22]. Therefore, targeting oxidative stress represents a promising therapeutic approach for hair loss.

Current treatments for hair loss use finasteride, minoxidil, and baricitinib. However, these treatments are associated with side effects such as decreased libido, impotence, and problems with ejaculation [23]. Consequently, research on natural products is emerging as a promising alternative treatment for hair loss [24,25,26]. *Rhus semialata* gall extract is a plant extract with many uses, such as skin care, oral care, and traditional medicine [27]. *Rhus semialata* gall has been previously reported to have antioxidant properties and protective effects in keratinocytes [27,28,29,30,31]. However, we found no research on the potential effects of *Rhus semialata* gall in inhibiting hair loss.

In this study, we extracted a water-soluble fraction of *Rhus semialata* gall extract (WRGE) through a standardized extraction process. Furthermore, we detected Penta-O-Galloyl-β-D-Glucose (PGG), a major component of WRGE, using HPLC-DAD analysis and identified its structure through HRESIMS/MS. We investigated the hair growth effects of WRGE and PGG. WRGE and PGG promoted the proliferation and expression of growth factors in HDPCs. Furthermore, when WRGE and PGG were applied to HDPCs treated with DHT and H_2_O_2_, which mimic hair loss-inducing conditions, anti-hair loss effects were observed. These findings were further supported by positive outcomes in human clinical trials. Collectively, these results suggest that WRGE and PGG have potential as novel therapeutic agents for the treatment of hair loss.

## 2. Materials and Methods

### 2.1. Preparation of WRGE

Dried galls of *Rhus semialata* were purchased from Fufeng Sinuote Bio-Tech Co., Ltd. (Baoji, China). A total of 200 g of galls was combined with 2 L of distilled water and extracted at 100 °C for 4 h. The extract was filtered using filter paper, and the resulting filtrate was concentrated under reduced pressure using a rotary evaporator. Subsequently, 95% ethanol was added to achieve a final concentration of 80% ethanol, with the volume being 12 times the weight of the solid content. The mixture was stirred at room temperature for 1 h to dissolve the extract, followed by filtration through filter paper. The filtrate was further concentrated under reduced pressure and vacuum-dried. The extraction yield was 52%.

### 2.2. HPLC and HRESIMS Analysis of WRGE

An Agilent HPLC 1200 series system equipped with an autosampler, column oven, pumps, DAD, and UV detector with a Phenomenex^®^ Luna C18 column (250 × 4.6 mm, 5 μm) was used for analysis (Phenomenex Inc., Torrance, CA, USA). Elution with solvent A (0.3% acetic acid in D.W.) and solvent B (0.3% acetic acid, 5% D.W in acetonitrile) in a gradient elution at a flow rate of 1 mL/min was carried out as follows: 0–15 min, 15–20% B; 15–20 min, 20–50% B; 20–25 min, 50% B; 25–25.1 min, 50–15% B; 25.1–30 min, 15% B. The detection wavelength was set at 280 nm. The column temperature was kept at 40 °C, and the injection volume of all samples was 10 μL.

HRESIMS detection was performed in the *m*/*z* range of 100–2000, and the resolution of the Orbitrap Exploris 120 (Thermo, Waltham, MA, USA) was fixed at 60,000 for the full MS scan and 15,000 for the data-dependent MS^n^ scan. The HESI ion source parameters were configured as follows: spray voltage, 3.5 kV; vaporizer temperature, 275 °C; ion transfer tube temperature, 320 °C; sheath gas flow rate, 50 L/min; auxiliary gas flow rate, 15 L/min; and sweep gas flow rate, 1 L/min. A normalized higher-energy collision dissociation (HCD) energy of 30% was applied for ion collision in the Orbitrap detector.

Approximately 5.0 mg each of Penta-O-Galloyl-β-D-Glucose (PGG; Sigma-Aldrich, St. Louis, MO, USA) standard compounds were placed in a 5 mL volumetric flask and dissolved with 80% methanol. Further calibration concentrations were prepared by diluting a standard solution with 80% methanol. The detector response was linear within the range of concentrations injected (PGG: 12.5~50 µg/mL).

### 2.3. DPPH Radical Scavenging Activity

The Blois method was modified to measure the radical scavenging activity of WRGE and PGG [32]. DPPH (Sigma-Aldrich, St. Louis, MO, USA), a reagent containing free radicals, was used, and ascorbic acid was used as a positive control for comparison. Different concentrations of WRGE, PGG, and a 0.2 mM DPPH reagent were combined and reacted for 10 min. Then, the absorbance was measured at 550 nm using a microplate reader.

### 2.4. Cell Culture

HDPCs were purchased from CEFObio (Seoul, Republic of Korea) and cultured in CEFOgro™ Human Dermal Papilla Growth Medium (CEFObio, Seoul, Republic of Korea) with 5% CO_2_ at 37 °C. Passage 3 to 6 HDPCs were used in in vitro cultivation and all the experiments in this study.

### 2.5. Cell Viability Assay

The cell viability of HDPCs treated with WRGE and PGG was assessed using the WST-8 assay. The HDPCs were seeded on a 96-well plate at 2 × 10^4^ cells/well and incubated at 37 °C for 24 h. Subsequently, they were treated with various concentrations of WRGE and PGG for 24 h. Subsequently, the WST-8 assay kit (10 μL/well) (BIOMAX, Seoul, Republic of Korea) was added to the cells and incubated with the cells for 4 h. Cell viability was analyzed using a microplate reader at 450 nm.

### 2.6. H_2_O_2_-Induced Antioxidant Capacity Assay

The cytotoxicity of H_2_O_2_-induced HDPCs with WRGE and PGG was assessed using a WST-8 assay kit. The HDPCs were seeded on a 96-well plate at 2 × 10^4^ cells/well and incubated with 250 μM H_2_O_2_ for 1 h. After incubation, the HDPCs were treated with various concentrations of WRGE and PGG for 24 h. Cells’ survival and toxicity were measured by a WST-8 assay, as described above. The antioxidant capacity was determined by comparing the cell viability of HDPC treated with the WRGE and PGG after oxidative stress.

### 2.7. Intracellular ROS Measurement

The cellular ROS level was measured using H_2_DCFDA (Sigma-Aldrich, St. Louis, MO, USA) and a previously modified method [33,34]. HDPCs were seeded in Coverglass bottom 6-well plates (SPL, Pocheon-si, Republic of Korea) and incubated for 24 h at 37 °C, 5% CO_2_. After incubation, the HDPCs were treated with various concentrations of WRGE and PGG for 24 h. Then, to induce intracellular ROS generation, the cells were treated with 1 mM H_2_O_2_ and incubated for 10 min in an incubator. After inducing intracellular ROS generation, the cells were stained with 10 μM DCFDA solution for 30 min in the incubator. ROS generation was determined by measuring dichlorofluorescein (DCF) using an LSM 980 with Airyscan 2 (Carl Zeiss, Oberkochen, Germany) at excitation wavelengths of 485 nm and emission wavelengths of 520 nm.

### 2.8. Quantitative Reverse Transcription-Polymerase Chain Reaction (qRT-PCR)

Total RNA was extracted from HDPCs using a TRIzol Reagent (Invitrogen, Waltham, MA, USA) and reverse transcribed to cDNA using PrimeScript™ RT reagent Kit (Takara, Tokyo, Japan). The levels of target mRNAs relative to GAPDH were measured via qRT-PCR on QuantStudio 5 (Applied Biosystems, Foster City, CA, USA) using EzAmp™ qPCR 2X Master Mix (ELPIS-BIOTECH, Daejeon, Republic of Korea). The primer sequences are shown in Table 1.

### 2.9. Western Blot

Total protein was isolated from HDPCs as previously described [34,35,36]. The protein concentration was measured using Pierce™ Bradford Protein Assay Kit (Thermo, Waltham, MA, USA). Equal amounts of proteins were separated by acrylamide gel electrophoresis and transferred to 0.45 μM PVDF membranes (Merck, Darmstadt, Germany) or 0.2 μM nitrocellulose membranes (Cytiva, Marlborough, MA, USA), which were blocked with Tris-buffered saline containing 0.1% Tween 20 (TBS-T) supplemented with 5% bovine serum albumin or 5% skim milk. The primary antibodies for VEGF (Cell Signaling Technology, Danvers, MA, USA), IGF-1 (Cell Signaling Technology), Basic FGF (Cell Signaling Technology), β-catenin (Cell Signaling Technology), GSK3β (Cell Signaling Technology), p-GSK3β (Cell Signaling Technology), p-p38 (Cell Signaling Technology), p38 (Cell Signaling Technology), p-AKT (Cell Signaling Technology), AKT (Cell Signaling Technology), p-ERK 1/2 (Cell Signaling Technology), ERK 1/2 (Cell Signaling Technology), BAX (Cell Signaling Technology), Bcl-2 (Cell Signaling Technology), and β-Actin (Cell Signaling Technology) were probed onto the PVDF membrane or nitrocellulose membrane overnight at 4 °C. All the primary antibodies were diluted at 1:1000 using 5% bovine serum albumin (BSA) in TBS-T solution. Then, the secondary antibody, diluted at 1:2000 in the 5% BSA or 5% skim milk in TBS-T solution, was incubated for 10 min at room temperature. To detect the target protein, the chemiluminescent substrate reacted with the horseradish peroxidase conjugated with the secondary antibody for 1 min at room temperature. Target protein bands were observed and captured by Viber Fusion Solo S (Viber Lourmat, Alençon, France) and were quantified and normalized using Evolution-capt Edge software, version 18.12 (Viber Lourmat).

### 2.10. DKK-1 ELISA

The HDPCs were seeded on a 96-well plate at 2 × 10^4^ cells/well and cultured with 5 μM DHT for 1 h in a CO_2_ incubator and cultured with WRGE and PGG for 48 h. Then, DKK-1 secretion levels in HDPCs’ supernatants were detected using DuoSet ELISA kit (R&D Systems, Minneapolis, MN, USA) according to the manufacturer’s instructions.

### 2.11. Clinical Trial

To evaluate the efficacy and safety of alleviation of hair loss symptoms of WRGE, a randomized, double-blinded, placebo-controlled clinical study was performed. The clinical trial was conducted at the KC Skin Research Center (Seoul, Republic of Korea), a certified facility specializing in dermatological and cosmetic efficacy studies (Clinical Trials Registration number: KC-240429-H1). This study was conducted in accordance with the Declaration of Helsinki, and the protocol was approved by the Ethics Committee of KC Skin Research Center (KCIRB-2024-0037) in March 2024. Participant recruitment began in **April 2024**, and the final follow-up assessments were completed in **October 2024**. Written consent was obtained from all subjects before this study in accordance with guidelines for cosmetics used for the alleviation of hair loss symptoms. Fifty-two subjects who were diagnosed with androgenetic alopecia were chosen for this study. Those who had undergone surgical treatment for hair loss, such as hair transplantation or scalp reduction, and those who had taken dutasteride or finasteride orally within the last 6 months were excluded from this study. A computer-generated randomization sequence was created using SPSS version 23.0 (IBM Corp., Armonk, NY, USA). Participants were randomly assigned to the intervention or control group at a 1:1 ratio. Shampoo containing 1% WRGE and placebo shampoo were treated on the scalp and hair area over 24 weeks (once daily). All study procedures were conducted in accordance with the clinical trial guidelines for functional cosmetics related to hair loss relief, as established by the Ministry of Food and Drug Safety (MFDS) of Korea.

#### 2.11.1. Evaluation Method

The primary efficacy evaluation was performed through hair density measurement using Folliscope 5.0 (LeadM Corporation, Suwon, Republic of Korea). The secondary efficacy evaluation was conducted through a visual evaluation by researchers and a subject survey.

#### 2.11.2. Hair Density Measurement (Phototrichogram)

To measure hair density, the area to be evaluated was first designated, and the hair was sheared to a certain area (about 1 cm^2^). After labeling with a hair dye (tattoo solution), the surface of the scalp was photographed. The scalp was taken with a Folliscope 5.0 at the designated test area before treatment and at 8 weeks, 16 weeks, and 24 weeks after treatment with the product. The total number of hairs in an area of 1 cm^2^ of the image was analyzed and used as a hair density result.

#### 2.11.3. Hair Photography and Researchers’ Visual Evaluations

Subjects were photographed for the test area at an angle of 45° (the line of bangs) and 90° (top of the head) before and at 8 weeks, 16 weeks, and 24 weeks after treatment with the product under the same conditions. Based on the judgment criteria for the extent of hair condition accompanying it, two dermatologists made visual evaluations. If there was a difference in the evaluation between the two researchers, a low level was chosen. Visual evaluation grades according to the degree of hair distribution are shown as follows: very good, +3; good, +2; a little better, +1; no change, 0; very bad, −3; bad, −2; and a little worse, −1. To analyze the consistency of the visual evaluation of the two experts, the intraclass correlation coefficient (ICC) was analyzed [37,38,39,40].

### 2.12. Statistical Analysis

All in vitro data are expressed as mean ± standard deviation (SD). Differences between control and treatment groups were evaluated using Prism 9 statistical analysis software (GraphPad, San Diego, CA, USA). Student’s *t*-tests were used to compare individual treatments with the control. *p*-values less than 0.05 were considered to be statistically significant. IBM SPSS Statistics 23.0 statistical analysis program (International Business Machine Corporation, Armonk, NY, USA) was used to determine the significance of the change in hair measurement before and after product treatment and in the area of placebo/1% WRGE treatment. Statistical analysis was performed after excluding the data of the dropout when a dropout occurred during the test, and the statistical significance was confirmed when the significance probability was *p* < 0.05 in the 95% confidence interval. Normality was assessed using the Shapiro–Wilk, and the homogeneity of variance was assessed using an independent sample *t*-test (or Mann–Whitney U test). Repeated measures ANOVA (or Friedman test) were conducted to determine whether there were differences according to measurement conditions within groups, and repeated measures ANOVA (or GEE) was conducted to determine whether there were differences between groups.

## 3. Results

### 3.1. Analysis of PGG in WRGE Using HPLC and HRESIMS

**Figure 1:**
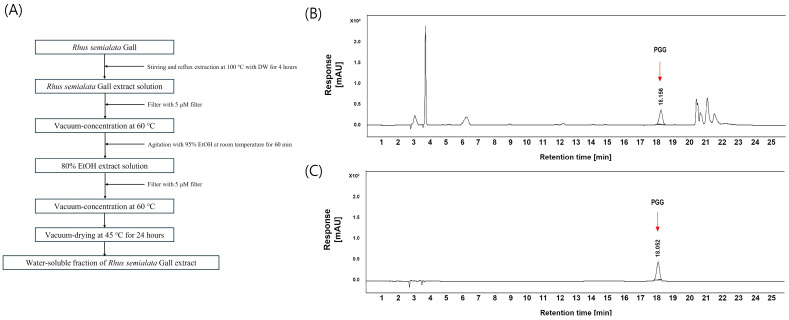
Scheme (**A**) showing process of extracting water-soluble fraction of *Rhus semialata* Gall extract. Representative high-performance liquid chromatography chromatogram at 280 nm: standard solutions of (**B**) WRGE, (**C**) PGG.

**Figure 2:**
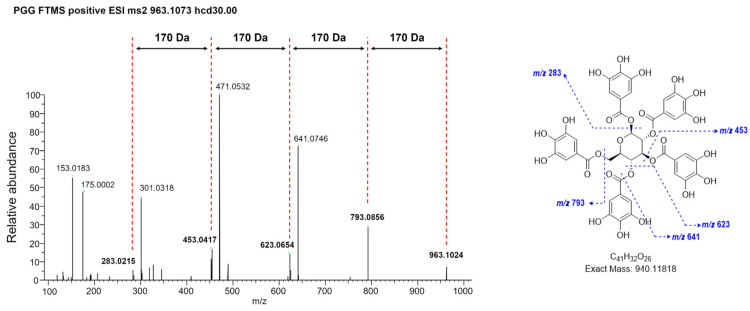
HRESIMS/MS fragmentation data of PGG (precursor ion *m*/*z* value of 963.1073).

WRGE was extracted from *Rhus semialata* gall as previously described (Figure 1A), and the extraction yield was 52%. The presence of PGG in WRGE was confirmed by comparison with an authentic PGG standard. HPLC-DAD analysis at 280 nm revealed a PGG peak in the WRGE sample with an RT of 18.16 min (Figure 1B), identical to that of the PGG standard (Figure 1C). Based on the absorption profile and the retention, PGG was identified as a major compound in the WRGE. The results confirmed that WRGE contained 9.7 ± 0.7% of PGG. In the HRESIMS/MS data of WRGE, PGG was detected with an *m*/*z* value of 963.1067 [M+Na]^+^, and its fragment ions of PGG were observed at *m*/*z* 793.0845 [963.1067—C_7_H_5_O_5_]^+^, 623.0590 [793.0845—C_7_H_5_O_5_]^+^, 453.0417 [623.0590—C_7_H_5_O_5_]^+^, and 283.0262 [453.0417—C_7_H_5_O_5_]^+^, corresponding to the sequential loss of galloyl (C_7_H_6_O_5_) units (Figure 2). These results confirm that the structure of PGG in WRGE is composed of five galloyl units, consistent with its known structural characteristics.

The content of PGG in WRGE was confirmed to be 9.7 ± 0.7% (*w*/*w*) using the established HPLC method. A series of calibration curves ranging from 62.5 to 1000 ppm demonstrated excellent linearity, with a correlation coefficient greater than 0.9996. The LOD and LOQ were determined to be 11.3 ppm and 34.1 ppm, respectively, based on the standard deviation of the linear response (σ) and the slope (S).

### 3.2. Hair-Inductive Properties of WRGE and PGG in HDPCs

**Figure 3:**
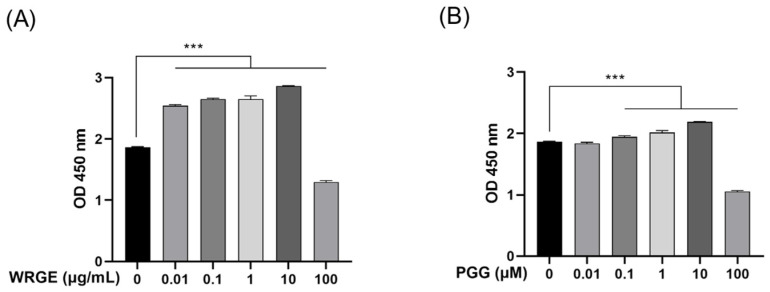
Effects of WRGE and PGG on cell viability in HDPCs. (**A**) HDPCs treated with indicated concentrations of WRGE for 24 h. (**B**) HDPCs treated with indicated concentrations of PGG for 24 h. Cell viability of HDPCs was measured via WST-8 assay. Results are presented as mean ± SD of three independent experiments and were analyzed with one-way ANOVA followed by Tukey’s test. *** *p* < 0.001.

The optimal concentrations of WRGE and PGG for the experiments were determined by measuring the cell viability of HDPCs at various concentrations of WRGE (0.01, 0.1, 1, 10, and 100 μg/mL) and PGG (0.01, 0.1, 1, 10, and 100 μM). WRGE showed toxicity at 100 μg/mL, and PGG showed toxicity at 100 μM. However, interestingly, cell proliferation was significantly increased at varying concentrations of WRGE (0.01, 0.1, 1, and 10 μg/mL) and PGG (0.1, 1, 10, and 100 μM) (Figure 3). Therefore, we selected three concentrations of WRGE and PGG for subsequent experiments, focusing on those that showed no toxicity and promoted cell proliferation (WRGE: 0.1–10 μg/mL, PGG: 0.1–10 μM).

**Figure 4:**
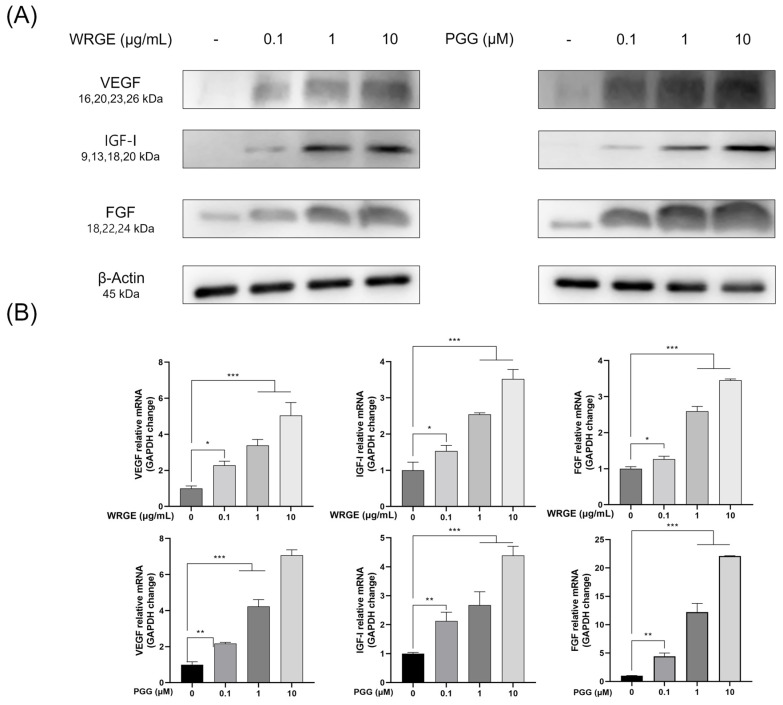
Effects of WRGE and PGG on hair-inductive properties in HDPCs. (**A**) Protein levels of VEGF, IGF-1, and FGF analyzed via Western blot while β-actin served as a loading control. (**B**) mRNA levels of VEGF, IGF-1, and FGF assessed via qRT-PCR and normalized against GAPDH. The qRT-PCR results are presented as the mean ± SD of three independent experiments and were analyzed with one-way ANOVA analysis followed by Tukey’s test. * *p* < 0.05, ** *p* < 0.01, and *** *p* < 0.001.

Hair growth factors, including VEGF, IGF-1, and FGF, play crucial roles in the growth and differentiation of HDPCs and regulate new hair formation [41,42]. HDPCs produce growth factors, including VEGF, IGF-1, and FGF. IGF-1 and FGF promote hair growth by inducing follicular tissue growth and the proliferation of hair follicle cells, and VEGF also enhances hair growth by stimulating angiogenesis, thereby supplying nutrients to hair follicle cells [43]. Therefore, we investigated whether WRGE and PGG affected the expression of hair-inductive factors. Treatment with WRGE and PGG significantly increased the protein expression levels of VEGF, IGF-1, and FGF in a dose-dependent manner (Figure 4A). Furthermore, WRGE and PGG significantly upregulated the mRNA expression levels of these growth factors in HDPCs (Figure 4B). These results indicate that WRGE and PGG promote the proliferation and expression of hair-inductive properties factors in HDPCs.

### 3.3. Effects of WRGE and PGG on the Wnt/β-Catenin Pathway and MAPK/AKT Pathway in HDPCs

**Figure 5:**
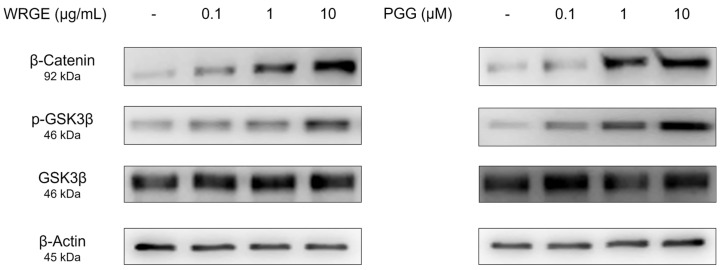
Effects of WRGE and PGG on Wnt/β-catenin signaling pathway. The protein level of β-catenin, p-GSK3β, and GSK3β analyzed via Western blot while β-actin served as a loading control.

**Figure 6:**
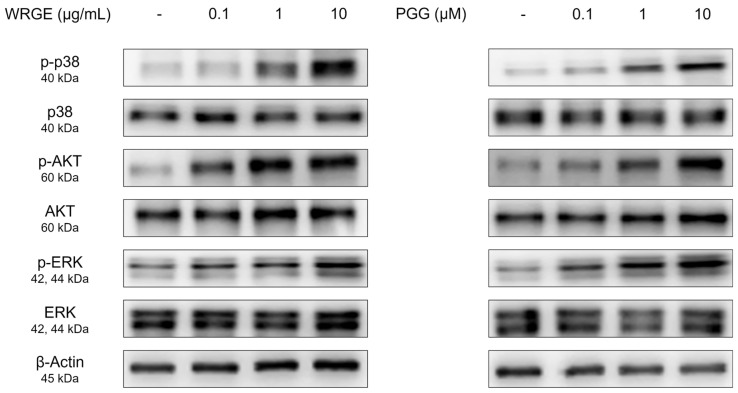
Effects of WRGE and PGG on MAPK/AKT signaling pathways. The protein level of p-p38, p38, p-AKT, AKT, p-ERK 1/2, and ERK 1/2 analyzed via Western blot while β-actin served as a loading control.

The Wnt/β-catenin signaling pathway regulates various physiological phenomena in HDPCs, including proliferation and hair growth factor production [44]. The binding of Wnt to frizzled receptors and a low-density lipoprotein-related protein causes the inactivation of glycogen synthase kinase-3β (GSK3β), which leads to the stabilization of β-catenin to avoid ubiquitin-dependent degradation [14,15]. β-catenin promotes the anagen state, hair-inductive factor secretion, and cell proliferation [45]. Consistent with enhanced proliferation and hair growth factor production in HDPCs, WRGE and PGG treatment significantly increased the Wnt/β-catenin pathway in GSK3β phosphorylation, along with a dose-dependent increase in β-catenin expression in HDPCs (Figure 5). We subsequently examined the upstream kinases responsible for phosphorylating the inhibitory site of GSK3β [14,15]. WRGE and PGG treatments significantly increased the phosphorylation levels of p38, AKT, and ERK in HDPCs (Figure 6). These results suggest that WRGE and PGG regulate the proliferation and production of hair growth factors in HDPCs by upregulating the Wnt/β-catenin and MAPK/AKT pathways.

### 3.4. WRGE and PGG Modulation of DHT-Induced DKK-1 and β-Catenin in HDPCs

**Figure 7:**
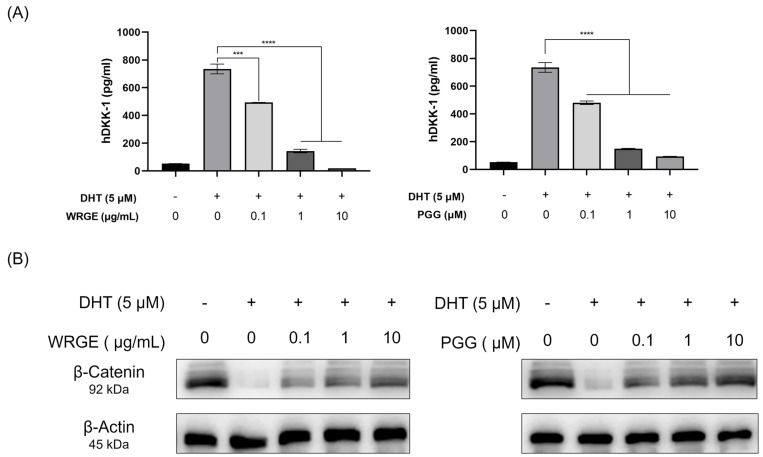
Effects of WRGE and PGG on DHT-induced secretion of DKK-1 and expression of β-catenin in HDPCs. (**A**) The protein level of DKK-1 analyzed via ELISA. (**B**) The protein level of β-catenin analyzed via Western blot while β-actin served as a loading control. The ELISA results are presented as the mean ± SD of three independent experiments and were analyzed with one-way ANOVA analysis followed by Tukey’s test. *** *p* < 0.001 and **** *p* < 0.0001.

The primary factor causing AGA is the conversion of testosterone, a male hormone, into DHT by 5α-reductase. When DHT binds to the androgen receptor, it induces the secretion of DKK-1, which leads to the death of hair cells and inhibits the Wnt/β-catenin signaling pathway, ultimately resulting in hair loss [46,47,48]. WRGE and PGG on HDPC treatment significantly inhibited the secretion of DKK-1, compared to the DHT-only group (Figure 7A). Consistent with these observations, the WRGE and PGG treatments significantly restored β-catenin expression in HDPCs exposed to DHT treatment (Figure 7B). These results show that WRGE and PGG treatment inhibited DKK-1 secretion in DHT-treated HDPCs through restoring β-catenin expression.

### 3.5. Antioxidant and Protective Effects of WRGE and PGG on H_2_O_2_-Induced Apoptosis in HDPCs

**Figure 8:**
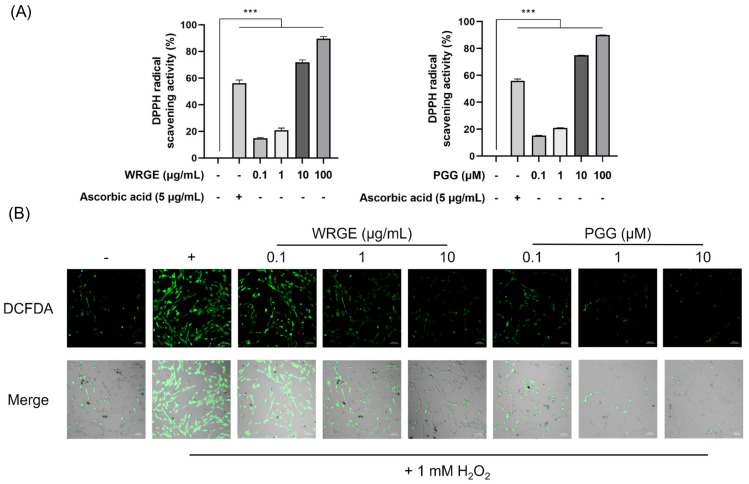
Antioxidant activities of WRGE and PGG. (**A**) Different concentrations of WRGE and PGG reacted with the DPPH solution for the indicated time. (**B**) Images of H_2_O_2_-induced DCFDA HDPCs captured by confocal microscopy. The DPPH assay results are presented as the mean ± SD of three independent experiments and were analyzed with one-way ANOVA analysis followed by Tukey’s test. *** *p* < 0.001.

Previous studies have demonstrated that excessive ROS generation and oxidative stress in hair tissue play a crucial role in the pathogenesis of alopecia [13,16,20,21,49]. Furthermore, HDPCs derived from balding scalps have exhibited increased sensitivity to oxidative stress. Under oxidative stress conditions, the inhibition of hair growth and the apoptosis of hair cells were observed due to the induction of premature senescence in HDPCs [17,22]. Consequently, we investigated whether WRGE and PGG exhibit antioxidant effects in vitro. WRGE and PGG showed DPPH radical scavenging activity in a dose-dependent manner (Figure 8A). To further determine whether WRGE and PGG could reduce intracellular ROS levels in HDPCs, a DCFDA assay was conducted. HDPCs were incubated with indicated concentrations of WRGE and PGG for 24 h. After incubation, 1 mM H_2_O_2_ was treated for 10 min to generate intracellular ROS. The results revealed a significant, dose-dependent decrease in DCF fluorescence intensity in the presence of WRGE and PGG compared to H_2_O_2_-treated HDPCs (Figure 8B). These findings suggest that WRGE and PGG exhibit antioxidant activity in vitro.

**Figure 9:**
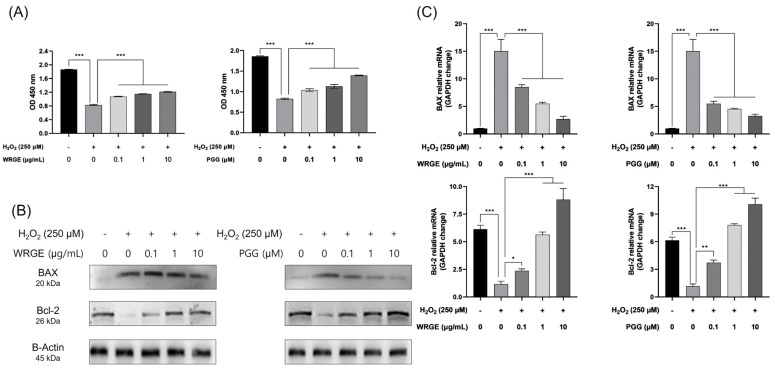
Protective effects of WRGE and PGG on H_2_O_2_-induced apoptosis in HDPCs. Reduced cytotoxicity of HDPCs treated with indicated concentrations of WRGE and PGG (**A**) against H_2_O_2_-induced apoptosis. HDPCs were incubated with WRGE and PGG for 24 h followed by treatment with 250 μM H_2_O_2_ for 1 h, with cytotoxicity being measured through a WST-8 assay. (**B**) Protein levels of BAX and Bcl-2 analyzed via Western blotting while β-actin served as a loading control. (**C**) The mRNA expression level of apoptosis-related genes (*BAX* and *BCL2*) was detected via RT-PCR, and *GAPDH* served as a loading control. The WST-8 assay and qRT-PCR results are presented as the mean ± SD of three independent experiments and were analyzed with one-way ANOVA analysis followed by Tukey’s test. * *p* < 0.05, ** *p* < 0.01, and *** *p* < 0.001.

Apoptosis in HDPCs is frequently observed in biopsies from hair loss patients, and hair follicle regression during the catagen phase is associated with apoptosis [50,51,52,53]. Excessive ROS production can be caused by aging, ultraviolet rays, and detailed haptics, which can induce apoptosis in HDPCs [16,18,19,20,21]. Based on the observation that WRGE and PGG exhibit antioxidant effects, we investigated whether these substances could protect HDPCs from H_2_O_2_-induced apoptosis. HDPCs were treated with WRGE and PGG after exposure to 0.2 mM H_2_O_2_ for 1 h, and cell viability was measured after 24 h. WRGE and PGG treatment significantly reduced cytotoxicity in a dose-dependent manner, compared to the H_2_O_2_-only group (Figure 9A).

BAX and Bcl-2 are crucial regulators of apoptosis. Bcl-2 functions as an inhibitor of apoptosis, whereas Bax acts as a promoter of apoptosis. The expression of these factors is tightly regulated and varies according to hair growth [54]. In this study, we treat H_2_O_2_ to induce apoptosis of HDPCs, which upregulated BAX and downregulated Bcl-2 [55,56]. However, treatment with WRGE and PGG reduced the protein expression of BAX while increasing the protein expression of Bcl-2 compared to H_2_O_2_-treated HDPCs (Figure 9B). Similarly, WRGE and PGG treatment decreased the mRNA expression of *BAX* and increased the mRNA expression of *BCL2* compared to H_2_O_2_-treated HDPCs (Figure 9C). These results indicate that WRGE and PGG may alleviate H_2_O_2_-induced apoptosis through the modulation of apoptosis-related factors in HDPCs.

### 3.6. Homogeneity Test

**Figure 10:**
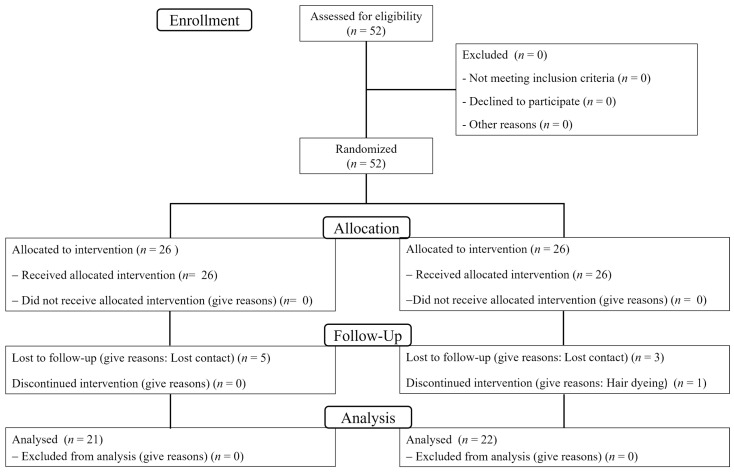
Clinical trial flow diagram.

A total of 52 subjects were enrolled and evenly allocated to the intervention and control groups (26 each), with age and sex balanced to maintain group comparability at baseline (Table 2). Of the total 52 subjects, 9 (4 in the test group and 5 in the control group) were lost to follow-up; finally, 43 participated in the study (Figure 10). The homogeneity of the “placebo” group and the “1% WRGE” group was checked. As a result of comparing the measured value of hair density before using the product, no significant difference between the two test groups was found (*p* = 0.743 > 0.05). Therefore, it was confirmed that these two groups were homogeneous (Table 3).

### 3.7. Measurement of Hair Density

#### 3.7.1. Measurement of Hair Density in the Placebo Treatment Group

**Figure 11:**
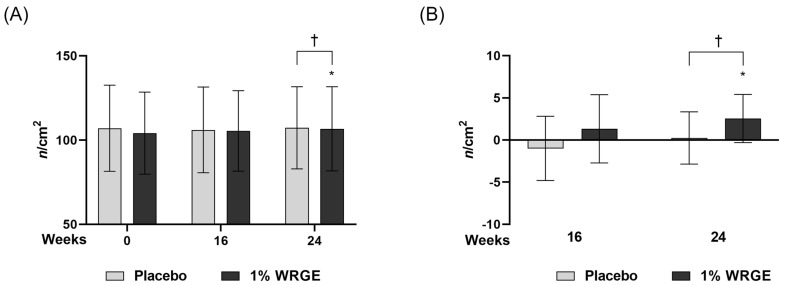
Comparing results of hair density between the “placebo” group and “1% WRGE” group. (**A**) Absolute hair density value at week 0, 16, and 24 and (**B**) the change percentage value in hair density. * *p* < 0.025, † *p* < 0.025.

The hair density of the placebo group decreased to −1.000 ± 3.808 *n*/cm^2^ (*p* = 0.243 > 0.05) after 16 weeks of treatment with the placebo and 0.238 ± 3.097 *n*/cm^2^ (*p* = 0.728 > 0.025) after 24 weeks of treatment with the placebo compared with that before treatment (Table 4, Figure 11). However, such decreases were not statistically significant.

#### 3.7.2. Measurement of Hair Density in the 1% WRGE Treatment Group

Hair density in the 1% WRGE treatment group significantly increased to 1.318 ± 4.052 *n*/cm^2^ (*p =* 0.020 < 0.05) after 16 weeks of treatment of 1% WRGE and 2.5450 ± 2.857 *n*/cm^2^ (*p =* 0.001 < 0.05) after 24 weeks of treatment of 1% WRGE compared with that before application. As a result of comparing the change in hair density between the placebo group and 1% WRGE group, there were signification differences after 24 weeks (*p =* 0.009 < 0.025) of treatment with the product (Figure 11, Table 5).

### 3.8. Visual Evaluation

#### 3.8.1. Researchers’ Visual Evaluations (ICC)

Visual evaluation was expressed as a score that evaluated the degree of hair distribution by two researchers (dermatologists), and the reliability of visual evaluation between Researcher 1 and Researcher 2 was analyzed using ICC. ICC is a value between 0 and 1, where less than 0.4 indicates poor reliability, 0.5–0.75 indicates medium reliability, 0.75–0.9 indicates good reliability, and greater than 0.9 indicates excellent reliability [24,37,38,39,40]. In the evaluation of the investigators in Table 6, Researcher 2 seemed to consistently give a higher score than Researcher 1; however, the degree of agreement between the two researchers’ visual evaluations at all times was 0.815 (Table 6).

#### 3.8.2. Researcher’s Visual Evaluation for the Placebo Treatment Group

**Figure 12:**
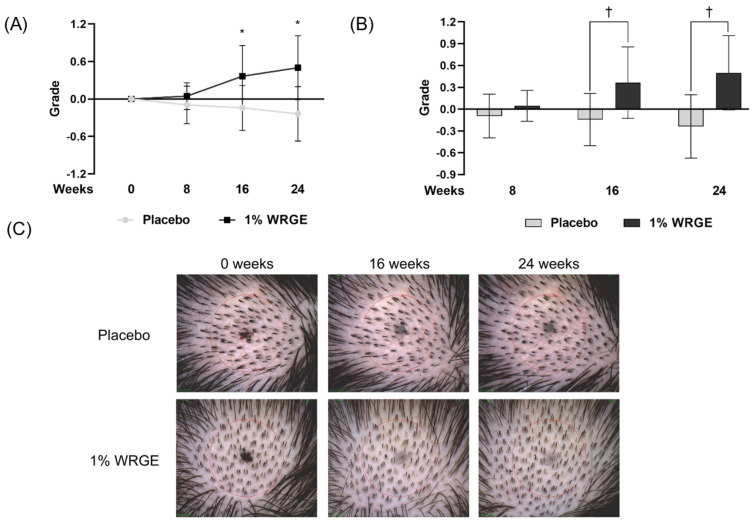
Visual evaluation results of the “1% WRGE” group compared with those of the “placebo” group. (**A**) Visual evaluation grade and (**B**) the changes in evaluation grade. (**C**) Images of change in hair density following the application of scalp shampoo for 24 consecutive weeks. * *p* < 0.017, † *p* < 0.017.

Visual evaluation scores of the two researchers for the placebo treatment group decreased to −0.095 ± 0.301 (*p =* 0.157) after 8 weeks of treatment with the placebo, −0.143 ± 0.359 (*p =* 0.083) after 16 weeks of treatment with the placebo, and −0.238 ± 0.436 (*p =* 0.025) after 24 weeks of treatment with the placebo compared with those before treatment (Figure 12, Table 6).

#### 3.8.3. Researchers’ Visual Evaluation for the 1% WRGE Treatment Group

The visual evaluation scores of researchers for the 1% WRGE treatment group increased to 0.045 ± 0.213 (*p =* 0.317 > 0.017) after 8 weeks of treatment with 1% WRGE compared with those before application (Figure 12, Table 7). However, the difference was not statistically significant. The visual evaluation scores of researchers for the 1% WRGE treatment group were significantly increased to 0.364 ± 0.492 (*p =* 0.005 < 0.017) after 16 weeks and 0.500 ± 0.512 (*p =* 0.001 < 0.017) after 24 weeks of treatment of 1% WRGE compared with those before treatment (Figure 12, Table 7).

Comparing the visual evaluation scores of researchers for the “placebo” group and the “1% WRGE” group, there were no significant differences after 8 weeks (*p =* 0.071 > 0.017), but there were significant differences after 16 weeks (*p =* 0.000 < 0.017) and 24 weeks (*p =* 0.000 < 0.017) of treatment with 1% WRGE (Table 7).

These clinical results show that the number of hairs, the primary efficacy evaluation, the expert visual evaluation score, and the secondary efficacy evaluation met the endpoint in the hair density change in the 1% WRGE group compared with the placebo group.

### 3.9. Safety Evaluation

The safety of the application site was evaluated in both the control and test groups at each visit through surveys and observations. As a result, no signs of irritation, including itching, pain, burning sensation, stinging, tightness, erythema, edema, or papules, were observed.

## 4. Discussion

This study investigated the potential of WRGE and its major component, PGG, in promoting hair growth and mitigating hair loss. *Rhus semialata* gall was extracted as WRGE according to the standardized process (Figure 1A). PGG was identified with HPLC-DAD and HRESIMS/MS. WRGE and PGG promote the proliferation and production of hair growth factors in HDPCs by upregulating the Wnt/β-catenin and MAPK/AKT pathways. Furthermore, WRGE and PGG inhibit the secretion of DKK-1 in DHT-treated HDPCs and exhibit anti-apoptotic effects in H_2_O_2_-treated HDPCs. These findings elucidate the molecular mechanisms underlying their efficacy, particularly in HDPCs, which are crucial for hair follicle regeneration. In clinical trials, a treatment containing 1% WRGE showed significant improvements in hair density and expert-assessed visual scores after 24 weeks of treatment.

The activation of the Wnt/β-catenin signaling pathway in HDPCs is a potential therapeutic strategy to inhibit hair loss [57,58,59]. In the present study, treatment with WRGE and PGG in HDPCs increases GSK3β phosphorylation, which decreases the ubiquitin-dependent degradation of β-catenin, leading to an increase in the expression of β-catenin (Figure 4). Furthermore, WRGE and PGG were found to enhance the MAPK/AKT signaling pathway (Figure 5), which regulates Wnt/β-catenin signaling. Wnt/β-catenin signaling is crucial in the expression of hair growth factors [8,9]. Therefore, WRGE and PGG treatment upregulate hair growth factors such as VEGF, IGF-1, and FGF in HDPCs (Figure 3). These results show that WRGE and PGG treatments enhance the production of hair growth factors in HDPC activity by upregulating the Wnt/β-catenin signaling pathway.

Angiogenesis plays a critical role in the hair cycle, particularly during the anagen phase, where it is highly active. During anagen, hair follicles require oxygen and nutrients supplied by blood vessels, leading to an increase in vascular density and active angiogenesis around the follicles. However, during catagen transition, angiogenesis decreases, resulting in reduced vascular density and diminished oxygen and nutrient supply to the follicles. Consequently, the follicles begin to regress, and apoptosis processes are triggered during catagen [60,61]. Angiogenesis is regulated by growth factors, including VEGF, IGF-1, and FGF. VEGF, secreted by HDPCs within the hair follicles, promotes vascular formation and prolongs the anagen phase [62]. IGF-1 suppresses apoptosis in follicular cells, thereby extending the anagen phase [63]. FGF becomes activated during anagen, increasing follicle size and density while also promoting the formation of new hair follicles through the activation of hair follicle stem cells [64]. In the present study, we showed that WRGE and PGG upregulated the expression of hair growth factors, including VEGF, IGF-1, and FGF, in HPDCs. These results demonstrate that WRGE and PGG may help hair growth through angiogenesis by increasing the expression of hair growth factors.

The major mechanism of hair loss caused by hormonal imbalance occurs when DHT, converted from testosterone by 5α-reductase, is produced excessively. The binding of DHT to HDPCs induces the secretion of DKK-1, which induces apoptosis of nearby HDPCs, leading to hair loss [65,66,67]. Moreover, it is known that DHT also inhibits Wnt/β-catenin signaling, leading to the ubiquitin-mediated proteolysis of β-catenin [9]. We observed that WRGE and PGG inhibit the secretion of DKK-1 by DHT treatment (Figure 7A) and restore the protein expression of β-catenin (Figure 7B). These results indicate that WRGE and PGG may inhibit DHT-induced hair loss by suppressing the secretion of DKK-1.

Oxidative stress is a known cause of tissue dysfunction and has been closely associated with hair follicle damage and hair loss [68,69,70]. Thus, this study aimed to clarify the antioxidant effects of WRGE and PGG on HDPCs. *Rhus semialata* gall is already known for its antioxidant and anti-inflammatory properties [28,29]. We also observed that WRGE and PGG showed radical scavenging activity (Figure 7A) and significantly decreased intracellular ROS levels in HDPCs (Figure 7B). Furthermore, oxidative stress-induced HDPC apoptosis is closely related to the pathogenesis of alopecia. Consistent with the antioxidant effect on HDPCs, WRGE and PGG treatments alleviate cytotoxicity in H_2_O_2_-induced apoptosis in HDPCs (Figure 8A). To explore the molecular mechanisms underlying these anti-apoptotic effects, the expression of apoptosis-related factors, including Bcl-2 and BAX, were analyzed. WRGE and PGG treatment led to significant reductions in the expression of pro-apoptotic BAX protein while increasing the expression of anti-apoptotic Bcl-2 protein compared to H_2_O_2_-treated HDPCs (Figure 8B). These results suggest that WRGE and PGG exhibited antioxidant properties, thereby mitigating oxidative stress-induced apoptosis in HDPCs.

The clinical results show that the 1% WRGE treatment group exhibited anti-hair loss efficacy. Hair densities in the 1% WRGE treatment group increased to 2.545 ± 2.5857 *n*/cm^2^ (*p =* 0.001 < 0.05) after 24 weeks of treatment compared to those before treatment (Figure 9A, Table 4). Furthermore, visual evaluation scores in the 1% WRGE treatment group increased to 0.500 ± 0.512 (*p =* 0.001 < 0.017) after 24 weeks compared to those before treatment. The findings of this study suggest that the test product may be effective in alleviating hair loss in adults with mild to severe symptoms over a 24-week period. Improvements in hair density and visual evaluation scores were observed without the occurrence of serious adverse events, indicating a favorable benefit-risk profile. Furthermore, the Wnt/β-catenin signaling pathway is also known to play a critical role in the proliferation and survival of hair follicle stem cells [71]. We suggest that WRGE treatment may enhance hair proliferation efficacy by activating Wnt/β-catenin signaling in HDPCs and hair follicle stem cells. The results of this clinical trial may be generalizable to adults in their 20s to 50s experiencing mild to severe hair loss. However, as this study was conducted at a single clinical site in Korea with a relatively homogeneous population, further research is needed to confirm the applicability of the findings to other ethnic groups or broader populations. The antioxidant and anti-inflammatory properties of *Rhus semialata* gall, used in traditional medicine, and its ability to protect keratinocytes have been reported in a previous study [28,29,31]. In this study, PGG was detected in the major peak of WRGE using HPLC-DAD. The structure of PGG was identified through HRESIMS/MS. Furthermore, we show that WRGE and PGG exhibit promising potential as natural therapeutic agents for hair growth and hair loss prevention. They act through multiple mechanisms, including activating Wnt/β-catenin signaling, suppressing DHT-induced DKK-1 secretion, and enhancing antioxidant defenses. In clinical trials, WRGE treatment on the scalp significantly increased hair density. Collectively, we investigated WRGE and PGG, showing the same anti-hair loss effects in HDPCs. These results suggest that PGG is an active compound in WRGE with an anti-hair loss effect. Future research should focus on their potential for use in combination therapies with current treatments such as minoxidil to enhance efficacy in treating hair loss.

## Figures and Tables

**Table 1:** Sequence primers used for qRT-PCR.

Gene	Primer Sequences (5′→3′)	Accession Number
VEGF	F: TCCTGGAGCGTGTACGTTG R: ACACGTCTGCGGATCTTGTA	NM_001171623.2
IGF-1	F: TCAACAAGCCCACAGGGTAT R: ACTCGTGCAGAGCAAAGGAT	NM_001111283.3
FGF	F: CTGTACTGCAAAAACGGGGG R: CGAAATGGAAATGAGGCGGA	X04431.1
BAX	F: GGGAGCAGCCCAGAGG R: ATTCGCCCTGCTCGATCC	NM_001291428.2
Bcl-2	F: GGATAACGGAGGCTGGGATG R: TTGTGGCTCAGATAGGCACC	NM_000633.3
GAPDH	F: ACTTTTGGTATCGTGGAAGGAC R: GCAGGGATGATGTTCTGGAG	NM_002046.7

**Table 2:** Clinical characteristics for each group.

Characteristic	1% WRGE Group	Placebo Group
Age (years) mean ± SD	46.3 ± 8.6	43.1 ± 7.6
Sex, *n* (%)		
Male	11 (42.3)	9 (34.6) 17 (65.4)
Female	15 (57.7)	

**Table 3:** Measurement results of hair density before treatment with the product in the “placebo” group and “1% WRGE” group.

Hair Density (Mean ± SD)		
Group Name	Total Hair Count (*n*/cm^2^)	*p*-Value ^a^
Placebo	107.048 ± 25.586	0.743
1% WRGE	104.136 ± 24.337	

Note. Shapiro-Wilk Normality Test—*p* < 0.05, compared between groups ^a^: comparison of placebo group and 1% WRGE group, *p* < 0.05 by Mann-Whitney U-test.

**Table 4:** Measurements of hair density in the “placebo” treatment group.

Hair Density (Mean ± SD)			
Measurement Time Point	*n*/cm^2^	Variation ^a^	*p*-Value ^b^
0 weeks	107.048 ± 25.586	-	-
16 weeks	106.048 ± 25.471	−1.000 ± 3.808	0.243
24 weeks	107.286 ± 24.350	0.238 ± 3.097	0.728

Note. Shapiro–Wilk Normality Test—*p* < 0.05, Variation ^a^: $(\sum_{k=0}^{a}Measure\;value\;of\;weeks\;after\;treatment\left(k\right)-Measured\;value\;before\;treatment\;(k))$/a, *p*-value ^b^: *p* < 0.025 Repeated measures ANOVA, post hoc Bonferroni correction.

**Table 5:** Measurement of hair density in the “1% WRGE” treatment group.

Hair Density (Mean ± SD)				
Measurement Time	*n*/cm^2^	Variation ^a^	*p*-Value	
			Compared Within Groups ^b^	Compared Between Groups ^c^
0 weeks	104.136 ± 24.337	-	-	-
16 weeks	105.455 ± 23.876	1.318 ± 4.052	0.166	0.048
24 weeks	106.682 ± 24.952	2.545 ± 2.857	0.001 *	0.009 †

Note. Variation ^a^: $(\sum_{k=0}^{a}Measure\;value\;of\;weeks\;after\;treatment\left(k\right)-Measured\;value\;before\;treatment\;(k))$/a, Shapiro-Wilk Normality Test (compared within groups)—*p* < 0.05, compared within groups ^b^: * *p* < 0.025 by Friedman test, post hoc Wilcoxon signed rank test with Bonferroni correction. Shapiro-Wilk Normality Test (compared between groups)—*p* < 0.05, compared between groups ^c^: comparison of “placebo” group and “1% WRGE” group, † *p* < 0.025 by GEE.

**Table 6:** Researchers’ visual evaluation.

	8 Weeks	16 Weeks	24 Weeks
Researcher 1	0.163 ± 0.485	0.349 ± 0.529	0.372 ± 0.691
Researcher 2	0.000 ± 0.309	0.116 ± 0.498	0.163 ± 0.574
ICC		0.815	

**Table 7:** Visual evaluation results for the “1% WRGE” treatment group.

Hair Density (Mean ± SD)				
Measurement Time	Grade	Variation ^a^	*p*-Value	
			Compared Within Groups ^b^	Compared Between Groups ^c^
0 weeks	0.000 ± 0.000	-	-	-
8 weeks	0.045 ± 0.213	0.045 ± 0.213	0.317	0.071
16 weeks	0.364 ± 0.492	0.364 ± 0.492	0.005 *	0.000 †
24 weeks	0.500 ± 0.512	0.500 ± 0.512	0.001 *	0.000 †

Note. Variation ^a^ = $(\sum_{k=0}^{a}Measured\;value\;of\;n\;weeks\;after\;treatment\left(k\right)-Measured\;value\;of\;before\;treatment\;(k))$/a, Shapiro–Wilk Normality Test (compared within groups)—*p* < 0.05, compared within groups ^b^: * *p* < 0.017 (5%/3) by Friedman test, post hoc Wilcoxon signed rank test with Bonferroni correction. Shapiro–Wilk Normality Test (compared between groups)—*p* < 0.05, compared between groups ^c^: comparison of “placebo” group and “1% WRGE” group, † *p* < 0.17 (5%/3) by GEE.

## Data Availability

The data presented in this study are available in the article.

## Abbreviations

The following abbreviations are used in this manuscript:

AGA	Androgenetic alopecia
BSA	Bovine serum albumin
DCFDA	2′, 7′–dichlorofluorescein diacetate
DHT	Dihydrotestosterone
DKK-1	Dickkopf-related protein 1
DPPH	2,2-diphenyl-1-picrylhydrazyl
ELISA	Enzyme-linked immunosorbent assay
FGF	Fibroblast growth factor
GSK3β	Glycogen synthase kinase 3β
H2O2	Hydrogen peroxide
HDPCs	Human dermal papilla cells
HF	Hair follicle
HPLC	High-performance liquid chromatography
HRESIMS	High-resolution electrospray ionization mass spectrometry
IGG	Intraclass correlation coefficient
IGF-1	Insulin-like growth factor 1
MAPK	Mitogen-activated protein kinase
PGG	Penta-O-Galloyl-β-D-Glucose
qRT-PCR	Quantitative reverse transcription-polymerase chain reaction
ROS	Reactive oxygen species
VEGF	Vascular endothelial growth factor
Wnt	Wingless
WRGE	Water-soluble fraction of *Rhus semialata* gall extract
